# Horizontal Transfer of Diatomaceous Earth and Botanical Insecticides in the Common Bed Bug, *Cimex lectularius* L.; Hemiptera: Cimicidae

**DOI:** 10.1371/journal.pone.0075626

**Published:** 2013-09-25

**Authors:** Yasmin Akhtar, Murray B. Isman

**Affiliations:** Faculty of Land and Food Systems, University of British Columbia, Vancouver, BC, Canada; United States Department of Agriculture, Beltsville Agricultural Research Center, United States of America

## Abstract

**Background:**

Horizontal transfer of insecticide occurs when insects contact or ingest an insecticide, return to an aggregation or a nest, and transfer the insecticide to other conspecific insects through contact. This phenomenon has been reported in a number of insects including social insects, however it has not been reported in bed bugs. Since horizontal transfer can facilitate the spread of insecticide into hard to reach spaces, it could contribute greatly to the management of these public health pests.

**Methodology/Results:**

To demonstrate horizontal transfer of diatomaceous earth and botanical insecticides in *C. lectularius*, an exposed (donor) bed bug, following a 10-minute acquisition period, was placed with unexposed (recipient) bed bugs. Mortality data clearly demonstrates that diatomaceous earth (DE 51) was actively transferred from a single exposed bug to unexposed bugs in a concentration dependent manner. LC_50_ values varied from 24.4 mg at 48 h to 5.1 mg at 216 h when a single exposed bed bug was placed with 5 unexposed bed bugs. LT_50_ values also exhibited a concentration response. LT_50_ values varied from 1.8 days to 8.4 days when a ‘donor’ bug exposed to 20 and 5 mg of dust respectively was placed with 5 ‘recipient’ bugs. Dust was also actively transferred from adult bed bugs to the nymphs. In addition we observed horizontal transfer of botanical insecticides including neem, ryania, and rotenone to varying degrees.

**Conclusion/Significance:**

Our data clearly demonstrate horizontal transfer of diatomaceous earth and botanical insecticides in the common bed bug, *C. lectularius*. Use of a fluorescent dust provided visual confirmation that contaminated bed bugs transfer dust to untreated bed bugs in harborage. This result is important because bedbugs live in hard-to-reach places and interaction between conspecifics can be exploited for delivery and dissemination of management products directed at this public health pest.

## Introduction

Horizontal transfer of insecticides occurs through the transfer of the active ingredients among individuals within an insect population through contact. The phenomenon occurs when the most active members of a colony, often foraging adults, become exposed to an insecticide residue, which is subsequently transferred to unexposed members of the colony upon returning to the nest. Several mechanisms have been described for this transfer including mutual grooming [1] trophallaxis [2] necrophagy [3] coprophagy [4] and emetophagy [5].

Horizontal transfer of pathogens (autodissemination) and insecticides have been reported in several insect orders, including Blattodea [6] Lepidoptera [7] Coleoptera [8], Diptera [9], [10] and social insects, *viz*. Isoptera and Hymenoptera [1], [11]. However, studies of horizontal transfer of insecticides in bed bugs are lacking.

Bed bugs (*Cimex lectularius* L.; Hemiptera: Cimicidae) have re-emerged as important public health pests in the past decade, with increasing intensity of urban infestations in North America, western Europe, Japan and Australia [12], [13], [14]. The exact cause of this resurgence is unclear, but may be a consequence of (i) the development of resistance in bed bugs [15], [16], [17] to commonly used domestic insecticides, (ii) increased human movement – both travel and migration, (iii) decreased public awareness, and (iv) global warming. Although, the consumer market is currently flooded with products of dubious composition and efficacy, the search for new active ingredients and innovative delivery tools continues to provide effective means of dealing with bed bugs, one of the most economically and medically important pests of the urban environment.

Although, the majority of bed bug control methods to date rely on direct application of insecticides [18] information regarding secondary mortality due to transfer of insecticide from exposed to unexposed individuals within the target population is lacking. Contact insecticides (*viz*. Sterifab™, Bedlam™) lacking residual activity against bed bugs require repeated applications to ensure they reach bed bugs, which remain hidden in crevices except when seeking and feeding on hosts. Subsequent applications also are needed to kill nymphs emerging from eggs, or bed bugs reintroduced into the habitat [19].

Since there are no reports on horizontal transfer of dusts or other insecticides in bed bugs, the main objective of our study was to investigate whether adults of *C. lectularius* exposed to diatomaceous earth and botanical insecticides could transfer them to unexposed members (adults and nymphs) of a population. A more thorough understanding of the influence of insecticides on bed bugs through horizontal transfer could form the basis for designing effective control strategies against bed bugs.

We have used a specific diatomaceous earth, DE 51, in our experiments owing to its strong toxic effects against the bed bug, *C. lectularius*, in laboratory bioassays (24-h LC_50_ and LC_95_ values = 0.24 and 0.95 mg respectively) through direct contact with the dust (Akhtar and Isman, unpublished data).

## Materials and Methods

### Samples (Dust and insecticides)

Diatomaceous earth, DE 51, produced by EP Minerals LLC, Reno, NV, was obtained from JP Textile Ltd., Vancouver, BC, and a luminous (fluorescent) powder kit #1162A was purchased from BioQuip Products Inc., Rancho Dominguez, CA.

A refined seed extract of *Azadirachta indica* ( = neem) (containing 30% azadirachtin) was provided by Fortune Biotech (Bangalore, India). Rotenone dust was purchased from Later Chemicals Ltd. (Richmond, BC. Canada) and ryania dust was a gift from Dr. Alan Knight (USDA ARS, Wenatchee, WA, USA).

### Study taxa

Bed sheets heavily infested with bed bugs of all stages were field-collected from low-income, multiple-dwelling buildings in urban Vancouver, BC. To the best of our knowledge, no insecticide had been applied to the premises. Bed bugs were transferred to glass jars using forceps. Up to 100 individuals were kept in a single glass container, under a 12∶12 LD photoperiod at 24±2°C and 65% relative humidity in the Insect Toxicology Laboratory of the University of British Columbia, Vancouver, BC. Insects were used in the experiments within 48 h of collection.

### Transfer trials

**Figure 1:**
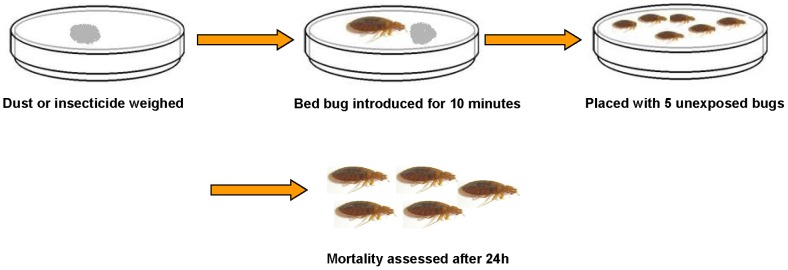
General experimental procedure.

General procedure (Figure 1): Samples were weighed in small plastic Petri dishes (Gelmon Sciences®; 5.0 cm diameter) lined with filter paper (Fisher Scientific®; 4.25 cm diameter) on the bottom. Adult bed bugs destined to be used as donors were introduced in Petri dishes containing the dust for 10 minutes. After the 10-minute acquisition period, the donor (exposed) individuals were then introduced into clean Petri dishes (Gelmon Sciences®; 5.0 cm diameter) along with the recipient (unexposed) bugs. Each Petri dish contained a filter paper (Fisher Scientific®; 4.25 cm diameter) folded in quarters serving as a harborage for the bed bugs. Mortality was assessed every 24 h. Controls were comprised of unexposed bed bugs only. Experiments were conducted at room temperature (23±2°C). At the end of the experiments digital images of representative insects were taken with an Olympus DSX 500 microscope for visual confirmation of the transfer of dust. Mortality among the receipient bed bugs was used as a measure of horizontal transfer of the insecticide.

### Horizontal transfer of DE 51 in *C. lectularius* through a single exposed bed bug

This experiment was conducted to demonstrate if a single bed bug exposed to DE 51 could transmit the dust to other unexposed bed bugs by sharing a harborage.

#### a. Determination of LC_50_ values

DE 51, red fluorescent dye (20 mg, 15 mg, 10 mg and 5 mg each) and a mixture of DE 51 and dye containing ½ the amount of dye and DE 51 in a 1∶1 ratio, for each concentration, were weighed in Petri dishes lined with filter papers followed by the introduction of an adult bed bug into each for a 10-minute acquisition period as described earlier. The exposed bed bug was then introduced in a Petri dish (5.0 cm diameter) containing 5 unexposed adult bed bugs. Petri dishes were placed in a plastic box with a lid. There were 5 replicates of 6 bugs each. Controls consisted of adult bed bugs, none of which were exposed to DE 51.

Mortality was assessed every 24 h for 10 days and was used as a measure of transfer. Dye was used in the experiment for visual confirmation of the transfer of DE 51 between exposed and unexposed bed bugs. LC_50_ values (concentrations needed to kill 50% of the bed bugs) were calculated for each time interval.

#### b. Determination of LT_50_ values

Time to kill 50% of the bed bugs was calculated for all groups as described in the previous section.

### Horizontal transfer of DE 51 to nymphs through exposed adult bed bugs

This experiment was conducted to demonstrate if DE 51 can be transferred from adult bed bugs to nymphs and was conducted the same way as the transfer between adults except that 4 exposed adult bed bugs were introduced into Petri dishes with 6 unexposed first or second instar nymphs. There were four replicates of 10 insects each. LC_50_ values were calculated for both adults and nymphs at 24 and 48 h intervals.

### Horizontal transfer of botanical insecticides through a single exposed adult bed bug

This experiment was conducted to demonstrate if different botanical insecticides could also be transferred like DE, therefore, DE 51 was used as a positive control in this experiment.

Twenty mg samples of neem powder (containing 30% azadirachtin as the active ingredient), ryania dust (containing approx. 1% ryanodine as the active ingredient), rotenone garden dust (containing 1% rotenone as the active ingredient) and DE 51 were weighed in Petri dishes lined with filter papers followed by the introduction of an adult bed bug into each for a 10-minute acquisition period as described earlier. One exposed adult bed bug was then introduced in a Petri dish (5.0 cm diameter) containing 5 unexposed adult bed bugs. There were 4 replicates of 6 bugs each. The control group consisted of 6 unexposed adult bed bugs. Mortality was assessed at 48, 96 and 144 h.

### Statistical analysis

LC_50_ values along with their corresponding 95% confidence intervals as well as LT_50_ values were calculated via Probit analysis using the EPA probit analysis program version 1.5. Four or five concentrations were used for each treatment (n = 3–4 replicates of 6–10 insect each). Data for the horizontal transfer of insecticides were analyzed by analysis of variance (ANOVA) using statistics software [20]. Where significant *F* values were found, Tukey's multiple comparison test was used to test for significant differences between individual treatments.

## Results

### Horizontal transfer of DE 51 in *C. lectularius* through a single exposed bed bug

#### a. Determination of LC_50_ values

Mortality of the recipient (unexposed) bed bugs placed with a DE 51- exposed bed bug was minimal for the first 24 hours but increased markedly thereafter. LC_50_ values were 24.4 and 5.1 mg at 48 h and 216 h, respectively (Table 1). Although the lowest LC_50_ value was observed at 216 h, it does not differ statistically from LC_50_ values calculated at 120 and 144 h, based on their overlapping confidence intervals (Table 1).

**Table 1:** Horizontal transfer of diatomaceous earth, DE 51 in *C. lectularius* adults through a single exposed bed bug.

	LC_50_ * (95% C.I)	
Time (h)		
	DE 51	dye + DE 51
48	>20.0	−
72	13.9 (9.5–30.2)	−
96	13.0 (7.5–43.9)	−
120	8.7 (4.6–12.1)	−
144	6.4 (2.8–8.7)	>20.0
216	5.1 (1.7–7.3)	17.6 (11.1–5134.2)

* LC_50_ values (concentration causing 50% mortality; in mg) were calculated based on 4 concentrations (5–20 mg) for each sample when an exposed bed bug was placed with 5 unexposed adult bed bugs.

− not calculated due to low mortality

There were 5 replicates of 6 bugs each. Exposed bed bug was excluded from the data (N = 25)

LC_50_ values for the dye were not calculated due to low mortality; there was no mortality in the control group.

Mortality of the unexposed bed bugs placed with a dye + DE 51 mixture-exposed bed bug was minimal (22%) until 96 h but increased markedly (55.6%) at 216 h. LC_50_ values of the unexposed bed bugs placed with a dye + DE 51 mixture-exposed bed bug were 28.9 and 17.6 mg at 144 and 216 h, respectively (Table 1).

**Figure 2:**
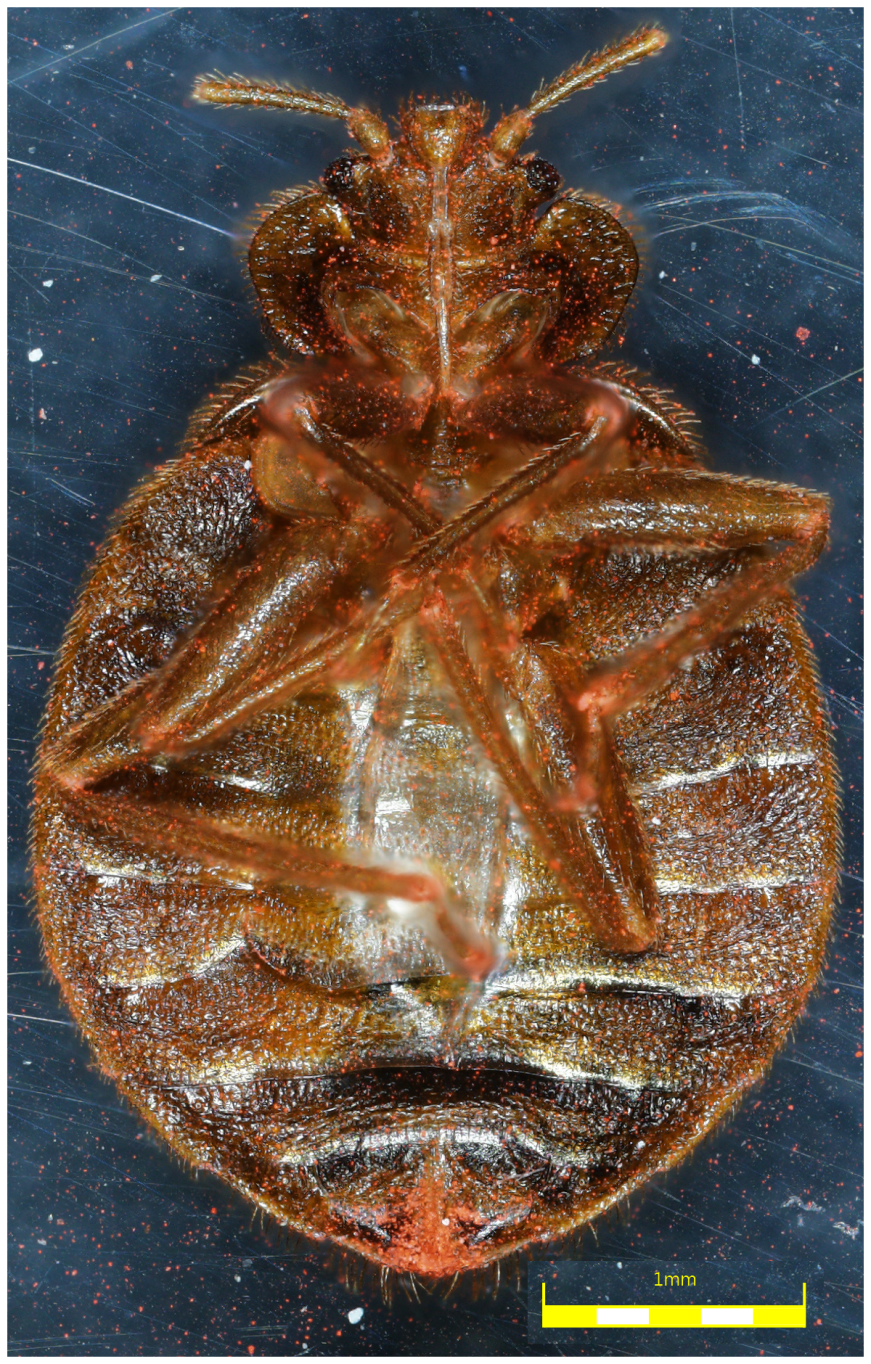
Adult bed bug, ventral view, showing accumulation of red dye particles around the bases of legs and distal tip of the abdomen.

Mortality was 10% in the unexposed bed bugs placed with a dye-only exposed bed bug (Table 1) at 216 h and no mortality occurred in the control group. The red fluorescent dye was visibly transferred from the single exposed bed bug to all others (Figure 2).

#### b. Determination of LT_50_ values

**Figure 3:**
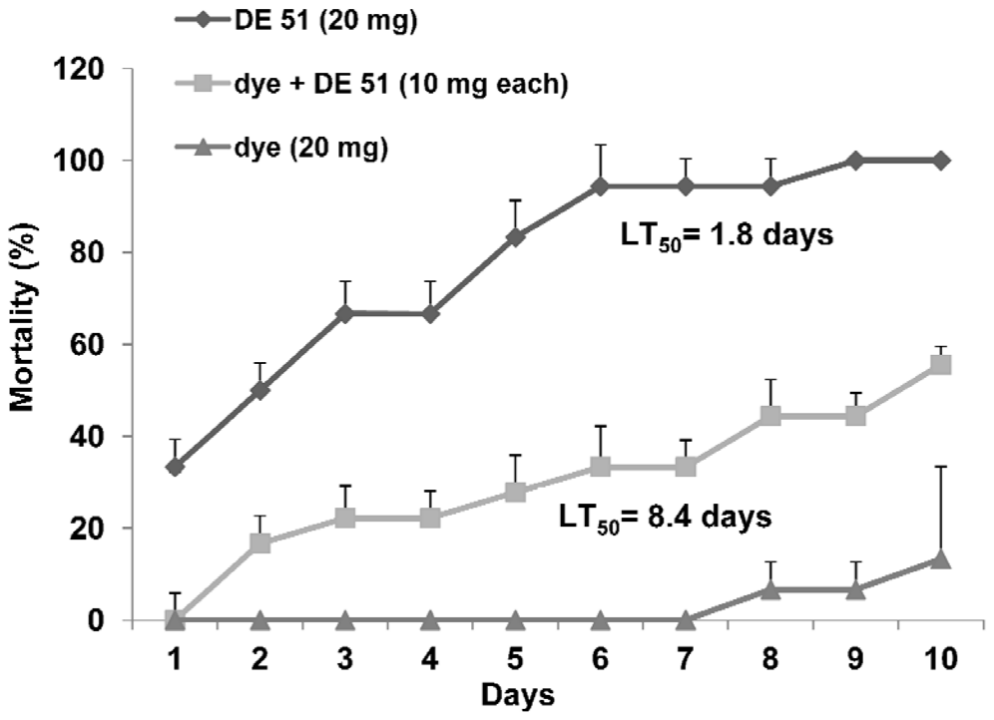
Horizontal transfer of DE 51 in *C. lectularius* through an exposed bug; one bed bug exposed to 20 mg of DE 51 or a mixture of DE 51 and the dye (10 mg each) or dye (20 mg) alone was placed with 5 unexposed bed bugs; LT_50_ = time required to kill 50% of the bugs. There was no mortality in the control group. There were 5 replicates of 6 bugs each. Exposed bed bug was excluded from the data; N = 25.

**Figure 4:**
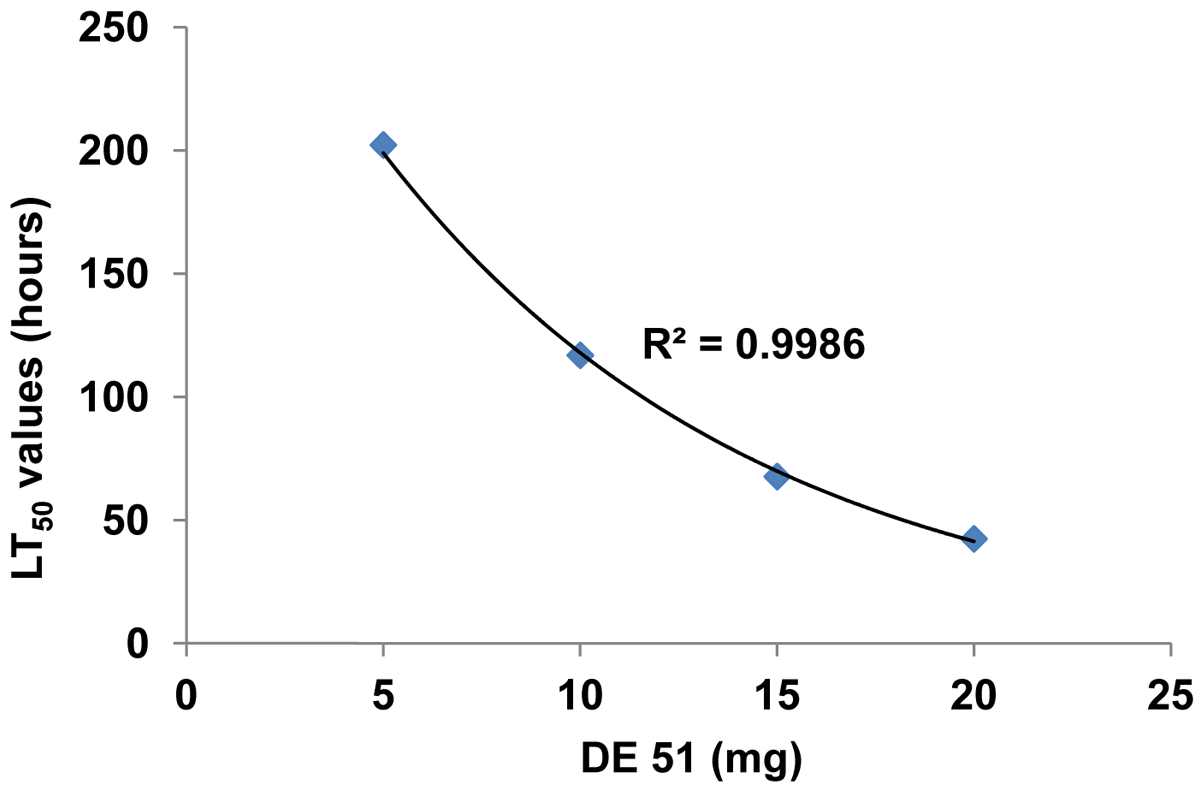
LT_50_ values of unexposed *C. lectularius* when placed with a bed bug exposed to different concentrations of DE 51 (5, 10, 15 and 20 mg). There were 5 replicates of 6 bugs each. Exposed bed bug was excluded from the data; N = 25.

Mortality of the unexposed bed bugs placed with DE 51- exposed bed bugs at different concentrations was calculated at different time intervals (24–216 h) as reported in the previous section and LT_50_ values (time to kill 50%) were calculated for each concentration. LT_50_ values along with 95% confidence intervals for the unexposed bed bugs placed with a DE 51- exposed bed bug ranged from 202.3 h (133.7–698.8) or 8.4 days at a concentration of 5 mg to 42.2 h (28.1–55.2) or 1.7 days at a concentration of 20 mg (Figures 3 and 4). The LT_50_ value for the unexposed adults placed with 20 mg DE 51-exposed bed bug was significantly different from all others except for the LT_50_ value at 15 mg. A plot of LT_50_ values versus different concentrations of DE 51 indicated a strong dose response (R^2^ = 0.9986) (Figure 4).

The LT_50_ value for unexposed bed bugs placed with a bed bug exposed to a 20 mg mixture of dye + DE 51 (1∶1) was 201.3 h (147.5–393.4) (8.4 days) (Figure 3). Mortality of the bed bugs placed with other concentrations of the mixture was too low (22–44.4% at 216 h) to calculate LT_50_ values. There was 13% mortality in the dye-only group and no mortality in the controls (Figure 3).

### Horizontal transfer of DE 51 to nymphs through exposed adult bed bugs

Mortality was high in nymphs at 24 h when DE 51-exposed adult bed bugs were placed with the unexposed nymphs. LC_50_ values for unexposed nymphs (8.1 mg) and unexposed adults (6.4 mg) did not differ significantly based on their overlapping confidence intervals (Table 2). Since 24 h mortality of the nymphs (55.6–94.4%) placed with 10–20 mg of DE 51- exposed adult bed bugs respectively and the mortality of the exposed bed bugs (66.7–100%) was greater than 50%, LT_50_ values were not calculated. High mortality rates within the first 24 h indicates that time to kill 50% of the nymphs and adults falls within the first 24 h.

**Table 2:** Horizontal transfer of diatomaceous earth, DE 51 to nymphs through exposed adult bed bugs.

		LC_50_ * (95% C.I)	
Treatments	Stage		
		24 h	48 h
dye	nymph	–	–
	adult	–	–
DE 51	nymph	8.1 (5.7–10.3)	**+**
	adult	6.4 (2.9–8.7)	**+**
dye+DE 51	nymph	19.9 (15.0–46.1)	8.9 (7.1–10.6)
	adult	–	11.8 (8.9–15.5)

* LC_50_ values (concentration causing 50% mortality; in mg) were calculated based on 4 concentrations (5–20 mg) for each sample when four exposed adult bed bugs were placed with six unexposed nymphs (1^st^–2^nd^ instar)

− not calculated due to low mortality

+ not calculated due to high mortality

There were 4 replicates of 10 bugs each. Mortality is based on a total of 24 nymphs and 16 adult bed bugs.

Mortality was considerably higher in nymphs when placed with dye + DE 51-exposed adults.

24 h LC_50_ value for nymphs was 19.9 mg, whereas there was no mortality in the adults (Table 2). There was a significant decline in the LC_50_ values of the nymphs at 48 h. LC_50_ values of the nymphs (8.9 mg) and adults (11.8 mg) at 48 h did not differ significantly based on their overlapping confidence intervals (Table 2).

There was no mortality in the adults or nymphs when dye-exposed adults were placed with nymphs. There was also no mortality in the control group (unexposed adults and nymphs) (Table 2).

### Horizontal transfer of botanical insecticides through a single exposed adult bed bug

**Figure 5:**
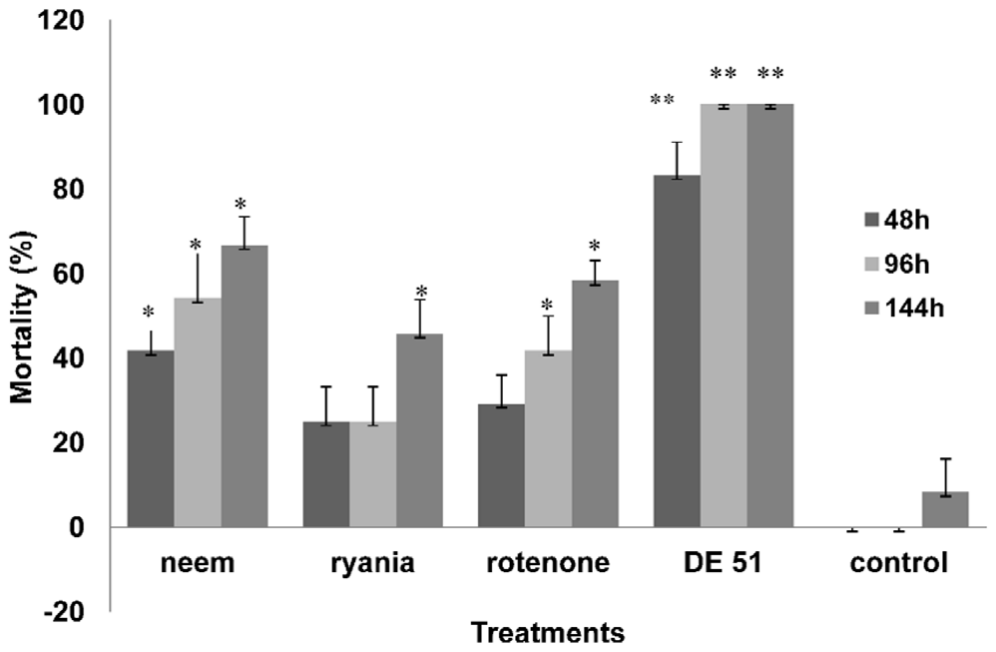
Horizontal transfer of DE 51, neem, ryania and rotenone in *C. lectularius* through an exposed bug; N = 4 replicates of 6 insects each; one bed bug with prior exposure to 20 mg of DE 51, neem, ryania and rotenone was placed with 5 untreated bugs; control insects were not exposed to the dust. There were 4 replicates of 6 bugs each. Exposed bed bug was excluded from the data (N = 20). ** Significantly different from all treatments including control. *Significantly different from control.

There was transfer of dust and botanical insecticides from a single exposed adult bed bug to unexposed adults (Figure 5). Mortality of the unexposed bed bugs was 83.3% (±8.3), 41.7% (±8.3), 24.9% (±6.8) and 29.2% (±7.9) when placed with a bed bug exposed to 20 mg of DE 51, neem, ryania, and rotenone, respectively at 48 h (Figure 5). A one-way ANOVA on the mortalities of different groups produced a significant F value (F_4,19_ = 18.8; P≤0.05). Mortalities of unexposed bed bugs placed with bed bugs exposed to DE 51 and neem were significantly greater than the control (Turkey's test: P≤0.05). There was no mortality in the control group.

Mortalities of the unexposed bed bugs at 96 h were greater than 48 h mortalities in all treatments except for the control (Figure 5). Mortalities of all groups except for ryania were significantly greater than the control (One-Way ANOVA; F_4,19_ = 23.5; Turkey's test; P≤0.05).

Likewise, 144 h mortalities of the unexposed bed bugs were greater than those at 96 h (Figure 5). At 144 h, mortalities of all groups were significantly greater than the control (One-Way ANOVA; F_4,19_ = 49.6; Turkey's test; P≤0.05). Mortality was <5% in the control group.

## Discussion

Our results have demonstrated that bed bugs contaminated with DE dust or botanicals caused mortality in other bed bugs while sharing a harborage through contact. Transfer of dust occurred not only between adults but also between adults and nymphs. The amount of dust the bed bug was exposed to prior to being placed with unexposed bed bugs seemed to play a major role in the time of transfer and the resulting mortality, as demonstrated by the concentration-response data. Use of a fluorescent dust provided visual confirmation that contaminated bed bugs transfer dust to unexposed bugs (Figure 2) in harbourage, confirming that bed bugs do not need to be in direct contact with pesticide residues to be affected. This result is important because bedbugs live in hard-to-reach places (cracks, crevices, picture frames, books, furniture) and as such interaction between the members of the colony can be exploited for delivery and dissemination of control products.

Horizontal transfer of insecticides in bed bugs is likely facilitated by their gregarious behavior, promoting contact between members and allowing for the rapid transfer of materials (dust or insecticides) from exposed to unexposed ones. Recent studies have demonstrated that aggregation of bed bugs is mediated by a combination of airborne [21] and contact pheromones [22], [23]. An airborne aggregation pheromone, composed of several short-chain aldehydes and monoterpenes occurring in the exoskeleton of immature bed bugs, has recently been shown to stimulate aggregation of adult and immature bed bugs in harborages when bugs are not foraging for hosts (e.g. during the photo phase). The chemical environment associated with a refuge (i.e., the presence of aggregation signals) has also been shown to be important for inducing aggregation in other blood feeding bugs such as a kissing bug [21]. In addition to pheromones, bed bugs are also known to be attracted to their feces and exuvia [22], [24].

Horizontal transfer of insecticide occurs when foragers contact or ingest an insecticide, return to the aggregation or nest, and translocate the insecticide to other members as well as the refuge and its vicinity. The crowded refuge conditions might be partially responsible for more frequent contacts with the insecticide-contaminated corpses, exuvium, faeces or parts of the contaminated substrate. Knowing that all stages of bed bugs (adults and immature) typically emerge from their aggregations at night to feed and return to their harborage before dawn [25], (personal observation) an effective approach to contaminate bed bugs during foraging would involve placing a toxicant in close proximity to their aggregations. This strategy has been very successful in social insects such as termites and ants as well as cockroaches, where a bait is offered to the foragers who in turn deliver the insecticide to the sedentary stages of the colony through contact, trophallaxis, coprophagy, and necrophagy [26]. Since aggregations are often located in inaccessible deep crevices and wall voids, a rapid dispersal of the members is required to increase contact with the toxicant. One way of achieving this is to augment these agents such as diatomaceous earth with the addition of bed bug alarm pheromones (*E*)-2-hexenal, (*E*)-2-octenal, and a (*E*)-2-hexenal:(*E*)-2-octenal blend) as has been demonstrated previously [27]. Addition of alarm pheromone to diatomaceous earth prompted a frenzied, rapid dispersal reaction in bed bugs, thereby promoting contacts with the toxicant. Elevating temperature can also be an effective strategy to increase insect movement and thereby increasing contact with a desiccant [28].

We have demonstrated horizontal transfer of DE 51 and other insecticides in our study. There was a progressive decrease in the LC_50_ values of DE 51 with time. Increased transfer of dust with time might have resulted from greater contacts between the bed bugs as well as with the contaminated substrate due to increased bed bug movement. Physiological conditions such as hunger or mating status are believed to be the driving forces initiating movement in bed bugs [29]. Since bed bugs were not fed during the course of the experiment, it is believed that search efficiency for the host may have increased with time. Time to kill 50% of the bed bugs was directly proportional to the amount of DE 51 the donor bed bug was exposed to. It took almost 4.8 times longer to kill recipient bed bugs when placed with a donor bed bug with prior exposure to 5 mg of DE 51 as opposed to 20 mg of dust.

Horizontal transfer of the dust was not affected by the life stage of the recipients. LC_50_ values of the donors and recipients were not significantly different from each other based on overlapping confidence intervals. Comparison of the mortality data (Tables 1 and 2) demonstrated that although a mixture of dye and dust (1∶1) was not effective in transfer between adult bed bugs regardless of the number of donors exposed, transfer was very effective between the donor adults and the recipient nymphs (Table 2) even in the first 24 h of exposure. However, the mixture was equitoxic to both the donors and recipients at 48 h (Table 2).

Our study has demonstrated an effective horizontal transfer of not only diatomaceous earth but also other dust-formulated botanical insecticides with different modes of action including neem, ryania and rotenone in *C. lectularius*. A significantly higher transfer of DE 51 compared with other insecticides may be based on its mode of action. Diatomaceous earth (DE) is usually regarded as an abrasive that scratches the cuticular surface, absorbs epicuticular wax and causes death through desiccation [30] Diatomaceous earth-based insecticides are finding increased use as stored commodity protectants, due to their nontoxic effects against humans and animals [30]. They have also been shown to be active against ticks [31], cowpea weevils [32], cockroaches, silverfish [33], ants [34] and numerous pests of grain [35].

We have demonstrated strong toxic effects of DE 51 against the bed bug, *C. lectularius*, in laboratory bioassays (24 h LC_50_ and LC_95_ values = 0.24 and 0.95 mg respectively) by directly exposing them to the dust (Akhtar and Isman, unpublished data). LT_50_ values for bed bugs directly exposed to 2 and 20 mg of DE 51, *viz*. 10 and 5 h respectively (Akhtar and Isman, unpublished data), indicate that at least 50% of the donor bed bugs will have more than sufficient time to reach a harborage for the effective transfer of toxin to other unexposed members of the colony before dying. The time to kill is inversely related to the amount of insecticide used, so there is a trade-off; selection of an intermediate dose of the insecticide will cause less mortality but would permit more bed bugs sufficient time to locate a harborage and transfer the toxin leading to secondary and tertiary mortality of conspecifics. Given that bed bugs stay in close contact with each other and release aggregation pheromones to help relocate their harborage after a blood meal [36], we believe that a contaminated bed bug will be well able to reach a harborage within several feet of the source.

Although, auto-dissemination of *Beauvaria bassiana* conidia (a microbial insecticide) via contact with contaminated individuals has been reported previously in bed bugs [37] there are no published studies on the horizontal transfer of dust and other botanical insecticides in bed bugs to our knowledge. Taking advantage of insect behaviour for designing novel control strategies can be an excellent approach to deal with bed bug infestations. Therefore development of different delivery systems allowing for maximum contact of bed bugs with the dust or other botanical insecticides will increase their impact through horizontal transfer facilitated by gregarious behavior of bed bugs. Future studies should focus on investigating this phenomenon in a field setting and against insecticide-resistant strains.
